# Electrospun Poly(L-lactide-co-ε-caprolactone) Nanofibers with Hydroxyapatite Nanoparticles Mimic Cellular Interplay in Bone Regeneration

**DOI:** 10.3390/ijms26115383

**Published:** 2025-06-04

**Authors:** Eva Šebová, Filipa Leal, Michala Klusáček Rampichová, Viraj P. Nirwan, Amir Fahmi, Pedro F. Costa, Eva Filová

**Affiliations:** 1 Department of Tissue Engineering, Institute of Experimental Medicine, Czech Academy of Sciences, 14200 Prague, Czech Republic; eva.sebova@iem.cas.cz (E.Š.); michala.rampichova@iem.cas.cz (M.K.R.); 2 Biofabics Lda, Rua Alfredo Allen 455, 4200-135 Porto, Portugalpedro.costa@biofabics.com (P.F.C.); 3 Faculty of Technology and Bionics, Rhine-Waal University of Applied Science, Marie-Curie-Straße 1, 47533 Kleve, Germany; virajpratap.nirwan@hochschule-rhein-waal.de (V.P.N.); amir.fahmi@hochschule-rhein-waal.de (A.F.)

**Keywords:** hydroxyapatite, osteoblasts, osteoclasts, PLCL, scaffold, bone regeneration, tissue engineering

## Abstract

This study investigates the impact of hydroxyapatite (HA) nanoparticles (NPs) on the cellular responses of poly(L-lactide-co-ε-caprolactone) (PLCL) scaffolds in bone tissue engineering applications. Three types of PLCL scaffolds were fabricated, varying in HANPs content. Saos-2 osteoblast-like cells (OBs) and THP-1-derived osteoclast-like cells (OCs) were co-cultured on the scaffolds, and cell proliferation was assessed using the MTS assay. The amount of double-stranded DNA (dsDNA) was quantified to evaluate cell proliferation. Expression levels of OBs and OCs markers were analyzed via quantitative polymerase chain reaction (qPCR) and the production of Collagen type I was visualized using confocal microscopy. Additionally, enzymatic activity of alkaline phosphatase (ALP) and tartrate-resistant acid phosphatase (TRAP or ACP5) was measured to assess OB and OC function, respectively. Interestingly, despite the scaffold’s structured character supporting the growth of the Saos-2 OBs and THP-1-derived OCs coculture, the incorporation of HANPs did not significantly enhance cellular responses compared to scaffolds without HANPs, except for collagen type I production. These findings suggest the need for further investigation into the potential benefits of HANPs in bone tissue engineering applications. Nevertheless, our study contributes valuable insights into optimizing biomaterial design for bone tissue regeneration, with implications for drug screening and material testing protocols.

## 1. Introduction

Bones are intricate structures in the human body, characterized by a complex interplay of various cell types responsible for bone formation and resorption. Osteoblasts, specialized bone-forming cells, play a crucial role in synthesizing the new bone matrix, which consists primarily of collagen fibers that provide structural support. These cells are also responsible for mineralizing the matrix, thereby contributing to bone density and strength [1]. Conversely, osteoclasts are specialized cells involved in bone resorption; they possess the unique ability to break down and dissolve the mineralized protein matrix within the bone, facilitating remodeling and maintenance of bone architecture [2].

The interaction between osteoblasts and osteoclasts is vital not only for maintaining physiological equilibrium between resorption and synthesis but also for effective bone healing processes. For instance, in joint replacement surgeries, the balance between osteoclast-mediated resorption and osteoblast-mediated synthesis is crucial for the integration and stability of implanted joint components, influencing both short-term healing and long-term joint function [3]. Similarly, during fracture healing, osteoblasts lay down new bone matrix at the site of injury while osteoclasts help clear away damaged tissue, allowing for the formation of callus and eventual bone union [4]. Optimizing this interplay is paramount to enhancing healing outcomes, accelerating recovery times, and ultimately reducing overall treatment costs.

The current market offers a variety of options tailored to different health conditions, defects, and patient circumstances. The gold standard for bone grafting is autografts, which utilize bone harvested from the patient’s own body due to their lower risk of rejection [5]. However, due to inherent limitations in availability, surgeons often resort to alternative options. Allografts involve tissue grafts sourced from different donors of the same species, thereby expanding the pool of available graft material. In contrast, xenografts utilize bone tissue from different species—commonly bovine or porcine sources—often processed into powder or ground pieces for surgical use [6,7,8,9]. Each alternative presents unique advantages and considerations based on patient needs and procedural requirements.

Recent advancements in polymer technology have led to significant innovations in scaffold design for bone tissue engineering. For instance, biopolymers such as collagen or gelatin scaffolds are widely tested in vitro and in vivo in various forms and their cytocompatibility and biodegradability were proven [10,11,12,13,14,15]. However, their mechanical properties are poor when used solely, so they are usually combined with compounds increasing stiffness and hardness [16,17,18]. It is not only nature-based polymers which are used in bone tissue engineering; synthetic ones offer versatility based on their structure degradation rates and their tunable properties [10,19]. Poly(lactic acid) (PLA) [20,21], poly(ε-caprolactone) (PCL) [22], poly(lactic-co-glycolic acid) (PLGA) [23,24,25], or polyethylene glycol [26] are utilized in bone tissue engineering due to their biocompatibility and/or biodegradability, supporting cell adhesion, proliferation and differentiation. Among these advancements is the use of copolymers such as poly(L-lactide-co-ε-caprolactone) (PLCL). This copolymer combines the favorable properties of both PLA and PCL—biodegradability and biocompatibility—while minimizing their individual limitations [27,28]. Electrospinning techniques have been employed to fabricate scaffolds from PLCL that mimic the fibrous architecture of natural bone extracellular matrix (ECM), promoting enhanced cell attachment and proliferation [27].

The incorporation of HA into PLCL scaffolds further enhances their osteoconductive properties. HA closely resembles the mineral component of natural bones and can form direct chemical bonds with living tissue, making it an ideal candidate for improving scaffold performance in bone regeneration applications [29,30,31]. Studies have shown that HA-modified PLCL scaffolds significantly improved cell metabolic activity and proliferation compared to non-modified counterparts [27].

The aim of this study is to investigate the suitability of Saos-2 cells as a model for OBs and THP-1-induced osteoclastogenesis as a model for OCs. By employing these cell models, we will demonstrate cellular responses to materials commonly used in bone-related research while emphasizing the importance of selecting appropriate cell models for material testing. This approach will provide valuable insights into the intricate interplay within bone tissue.

## 2. Results

### 2.1. Fibers Fabrication

**Figure 1:**
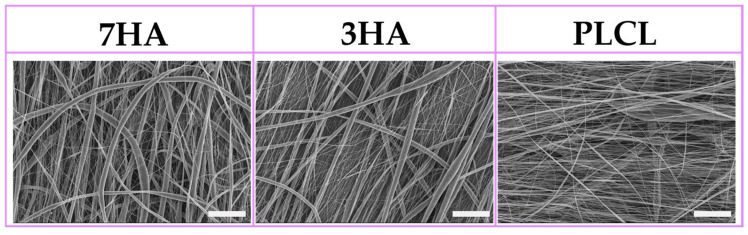
Representative SEM images of the different electrospun samples with 7% (7HA), 3% hydroxyapatite (3HA) or pristine PLCL. All images were acquired at a magnification of 400; scale bars: 50 μm.

The morphology and fiber size distribution of PLCL, 3HA, and 7HA scaffolds were analyzed using SEM (Figure 1). PLCL pristine fibers formed semi-aligned, beadless mats with a heterogeneous fiber size. In contrast, 3HA and 7HA displayed rough, porous fibers in non-woven mats with few beads. Additional details on material characteristics such as fiber diameter, chemical interactions between PLCL and HANPs, and contact angles analysis are available in the previously published study [27].

### 2.2. Metabolic Activity and Proliferation

**Figure 2:**
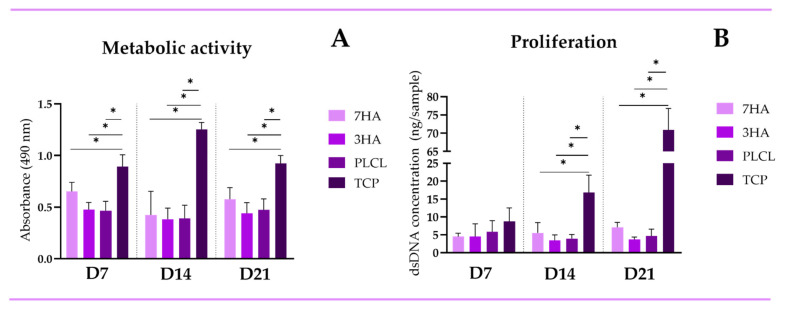
The metabolic activity (**A**) and proliferation (**B**) of THP-1 and Saos-2 cells coculture seeded on electrospun nanofiber scaffolds with 7% (7HA), 3% hydroxyapatite (3HA), pristine PLCL, and TCP. Data are presented as mean ± standard deviation (SD). The significant difference between groups is indicated by * (*p* < 0.05).

The metabolic activity of co-cultured THP-1 and Saos-2 cells was assessed using the MTS assay on days 7, 14, and 21 (Figure 2A). Cells seeded on tissue culture plastic (TCP) exhibited significantly higher metabolic activity compared to those seeded on electro-spun scaffolds at each time point (*p* < 0.05). Notably, while metabolic activity levels on scaffolds increased over time, there were no significant differences among the scaffold groups on any experimental day. The most pronounced difference in metabolic activity was observed on day 14. Cell proliferation was quantified using the PicoGreen assay, which measures the amount of double-stranded DNA (dsDNA) present in the samples. As shown in Figure 2B, there were no significant differences in dsDNA levels among the groups on day 7. However, by day 14, TCP samples demonstrated a significantly higher dsDNA content compared to all scaffold groups (*p* < 0.05), a trend that continued through day 21. On average, TCP samples contained approximately 70 ng/sample of dsDNA, while scaffolds exhibited dsDNA levels that were 10 to 19 times lower.

### 2.3. Enzymatic Activity

**Figure 3:**
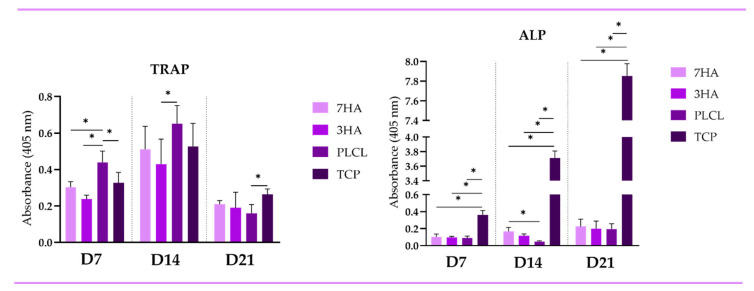
The tartrate-resistant acid phosphatase activity (osteoclastic marker) alkaline phosphatase activity (osteoblastic marker) of THP-1 derived osteoclasts and osteoblast-like Saos-2 cells in coculture seeded on electrospun nanofiber scaffolds with 7% hydroxyapatite (7HA), 3% hydroxyapatite (3HA) or pristine PLCL and tissue culture polystyrene (TCP). TRAP for osteoclast-like cells derived from THP-1 and ALP for Saos-2. Data are presented as mean ± standard deviation (SD). Significant difference between groups is indicated by * (*p* < 0.05).

The enzymatic activity of tartrate-resistant acid phosphatase (TRAP) and ALP was evaluated on days 7, 14, and 21 (Figure 3). On day 7, TRAP activity was significantly higher in cells seeded on PLCL scaffolds compared to those on other materials. By day 14, TRAP activity increased across all samples but remained highest in the PLCL group, with a significant difference noted when compared to the 3HA group (*p* < 0.05). On day 21, TRAP activity decreased in all samples; however, it was notably lowest in the PLCL group when compared to TCP. In contrast, ALP activity showed a significant increase in cells cultured on TCP from day 7 to day 21 (*p* < 0.05). On day 14, ALP activity was also elevated in cells cultured on scaffolds with 7% HA compared to those on pristine PLCL.

### 2.4. Quantitative PCR

#### 2.4.1. Osteoblastic Markers

**Figure 4:**
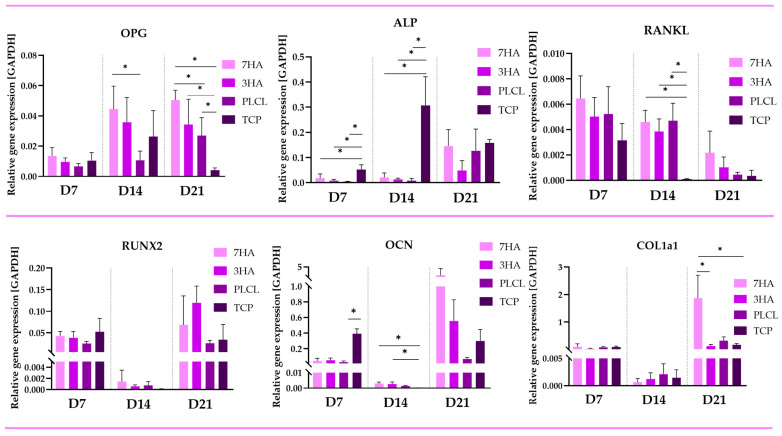
Relative gene expression of osteoblastic markers of osteoblast-like cells in coculture of Saos-2 and THP-1 derived osteoclast-like cells seeded on electrospun nanofiber scaffolds with 7% hydroxyapatite (7HA), 3% hydroxyapatite (3HA) or pristine PLCL and tissue culture polystyrene (TCP). Data are presented as mean ± standard deviation (SD). Significant difference between groups is indicated by * (*p* < 0.05).

The relative expression levels of osteoblastic markers involved in osteoclastic regulation—OPG, RANKL—and in osteogenic differentiation—ALP, RUNX2, OCN and COL1-a1—were assessed using qPCR on days 7, 14, and 21 (Figure 4). OPG expression was the lowest on day 7 across all groups. However, by day 14, a significant increase in OPG expression was observed in cells cultured on scaffolds containing HANPs, while no significant change was noted in cells seeded on pristine PLCL. Additionally, OPG expression also increased in cells on tissue culture plastic (TCP). On day 21, high levels of OPG expression persisted in cells seeded on scaffolds with HANPs, and a notable increase was also observed in cells on PLCL, unlike the cells on TCP with the lowest (*p* < 0.05) OPG ex-pression. RANKL expression showed variable trends but generally decreased over time, particularly in scaffolds with HANPs.

ALP expression consistently increased across all groups over the culture period, indicating ongoing osteoblastic activity. Specifically, the expression on days 7 and 14 was significantly higher in cells seeded on TCP. However, on day 21, no significant differences were observed among the groups. The graphs show that on day 7, there are no significant differences in the expression of RUNX2, and COL1a1 among the groups (7HA, 3HA, PLCL, and TCP). OCN expression is higher (*p* < 0.05) on day 7 significantly compared just to PLCL group. By day 14, all three markers exhibit a decrease in expression across all groups; however, OCN expression is significantly higher (*p* < 0.05) in cells cultured on 7HA and 3HA compared to TCP. On day 21 (D21), the expression levels of all three markers increase in all groups. While there are no significant differences between the groups for RUNX2 and OCN at this time point, COL1a1 expression is significantly higher (*p* < 0.05) in cells seeded on 7HA compared to both 3HA and TCP.

#### 2.4.2. Osteoclastic Markers

**Figure 5:**
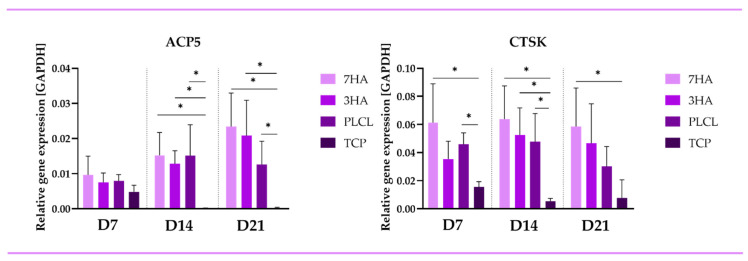
Relative gene expression of osteoclastic markers of osteoclast-like cells derived from THP-1 cells in coculture with Saos-2 seeded on electrospun nanofiber scaffolds with 7% (7HA), 3% hydroxyapatite (3HA) or pristine PLCL and tissue culture polystyrene (TCP). Data are presented as mean ± standard deviation (SD). Significant difference between groups is indicated by * (*p* < 0.05).

The expression levels of osteoclastic markers—cathepsin K (CTSK) and (TRAP)—were also analyzed via qPCR (Figure 5). ACP5 expression was significantly elevated in THP-1-derived osteoclast-like cells cultured on PLCL scaffolds compared to other conditions at all time points measured. On day 7, no significant differences were observed among the samples. From day 14, the expression of TRAP was increased in cells seeded on all nanofibers, compared to TCP. CTSK expression mirrored the trends of TRAP, further highlighting the dynamic interplay between osteoblast-like cells and osteoclast-like cells throughout the culture period in different environments.

### 2.5. Cell Visualization

**Figure 6:**
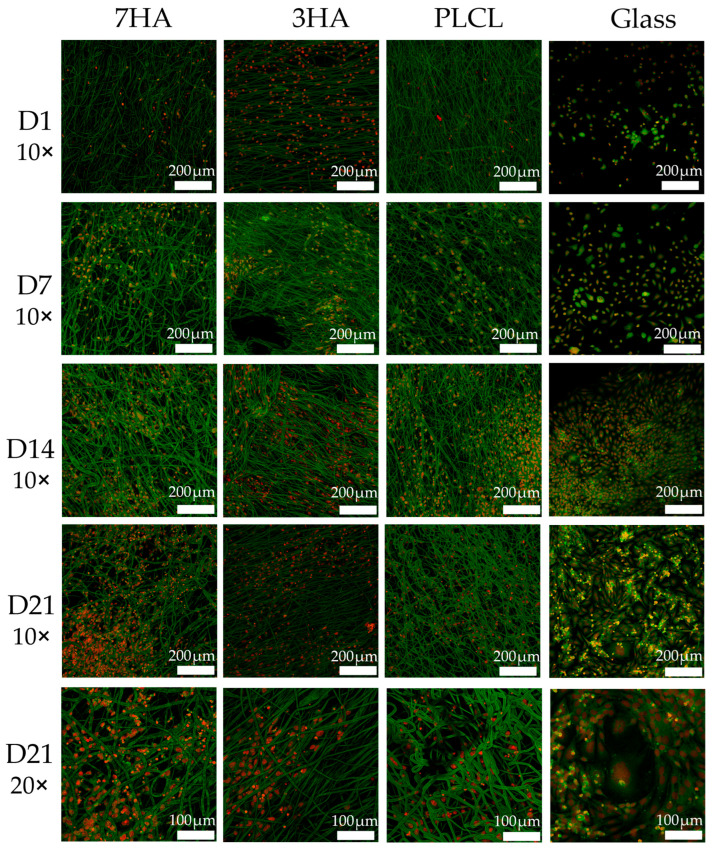
Distribution of cells in coculture seeded on electrospun nanofiber scaffolds with 7% hydroxyapatite (7HA), 3% hydroxyapatite (3HA) or pristine PLCL and coverglass on days 1, 7, 14 and 21. Propidium iodide—red, DiOC6(3)—green, scale bar 200 μm, 100 μm; objectives 10× and 20×.

Figure 6 displays the distribution of THP-1-derived osteoclast-like cells and Saos-2 osteoblast-like cells on electrospun PLCL scaffolds, visualized using confocal microscopy after dual staining with the fluorescent probe DiOC6(3) and propidium iodide (PI). The time points assessed include days 1, 7, 14, and 21. DiOC6(3) stains the intracellular membranes of cells, allowing for clear visualization of cell morphology and distribution on the scaffolds. The images reveal a dense population of viable cells adhering to the fibrous structure of the scaffolds, and PI staining for cell nuclei. Importantly, the fibers of the PLCL scaffolds are also visible in the images due to their interference with Di-OC6(3) that affected the clarity of the staining. Careful imaging settings were employed to minimize background fluorescence and enhance the visibility of both the cells and scaffold architecture. Overall, this figure illustrates the successful adhesion and distribution of cells on the scaffolds throughout the culture period.

### 2.6. Collagen Type I Staining

**Figure 7:**
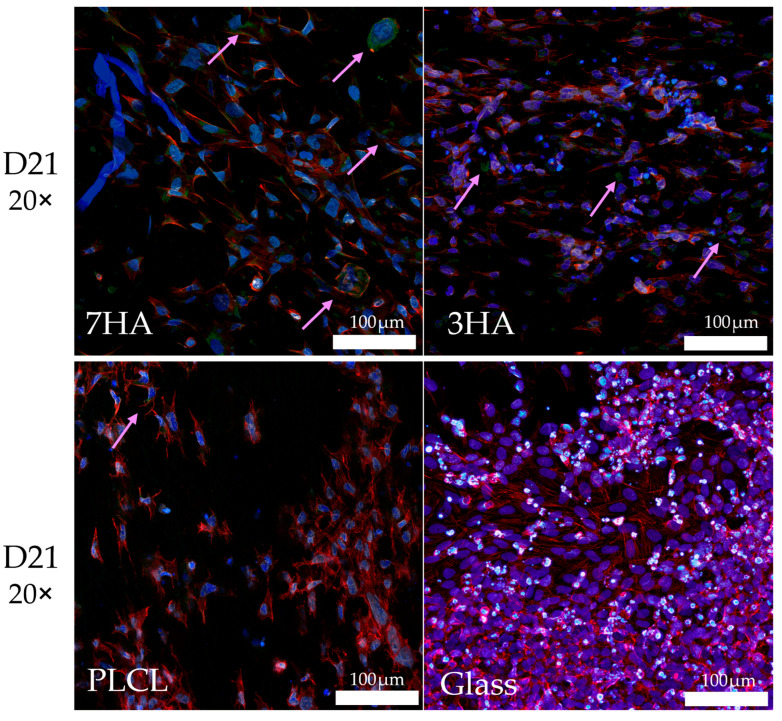
Representative micrographs of collagen type I produced by SaOS-2 in coculture with THP-1 derived osteoclasts cultured on electrospun nanofiber scaffolds with 7% (7HA), 3% hydroxyapatite (3HA), or pristine PLCL and cover glass. The pink arrows indicate regions where collagen type I is expressed. Scalebar: 100 μm; objective: 20× (green—collagen type I, red—actin, blue—nuclei).

The confocal scanning image (Figure 7) demonstrates the distribution of collagen type I produced by Saos-2 cells in a co-culture with THP-1-derived osteoclasts seeded on electrospun PLCL scaffolds, analyzed at day 21 (D21) of the co-culture period. Immunostaining reveals distinct differences in collagen type I deposition among the various scaffold types. The most extensive and intense green fluorescence, indicative of collagen type I, is observed on the 7HA scaffold, suggesting this substrate supports the highest level of collagen synthesis. Collagen type I is also detectable on the 3HA and PLCL scaffolds, although the signal on PLCL is sparse and barely discernible. In contrast, no collagen type I signal is present in cells cultured on coverglass. It is important to note that the fluorescence signal was not quantitatively measured, and the images are presented as representative qualitative observations without statistical analysis.

## 3. Discussion

The interplay between osteoblasts and osteoclasts is crucial for maintaining bone homeostasis, and the design of biomaterials that effectively support this interaction is essential for successful bone tissue engineering. In this study, we investigated the effects of HANPs incorporated into PLCL scaffolds on the cellular responses of Saos-2 osteoblast-like cells and THP-1-derived osteoclast-like cells. This study investigated a cell model consisting of Saos-2 cells and osteoclast-like cells derived from THP-1 to mimic the physiological conditions of the bone.

The metabolic activity of Saos-2 cells reached similar levels during the weeks of culture on the electrospun scaffolds containing HANPs, with no significant differences in averages. In contrast, metabolic activity on tissue culture plastic (TCP) was significantly increased on every experimental day. These findings align with previous studies indicating that while TCP supports rapid cell growth, it may not provide the optimal environment for osteogenic differentiation compared to 3D scaffolds enriched with bioactive materials [32,33,34,35]. Considerably similar results were obtained in ALP activity, where there was a notable rise (*p* < 0.05) of ALP in cells seeded on TCP from day 7 to day 21. This increase in ALP activity on TCP suggests an overgrowth of Saos-2 cells, consistent with findings that rapid proliferation can occur in flat cultures but may limit differentiation potential of THP-1 derived osteoclasts, based on low levels of osteoclastic genes expression [27,36]. Additionally, on day 14, ALP activity was higher in cells seeded on scaffolds containing 7% HANPs compared to those on PLCL alone. This result underscores the osteogenic properties of HANPs incorporated into the scaffolds and confirms outcomes from our previous study that demonstrated enhanced osteoblastic activity in HA-enriched environments [27,37,38,39,40]. The combination of increased ALP activity and DNA quantification suggests that the scaffolds not only support cell viability but also promote a more favorable microenvironment for osteogenic differentiation. The proliferation and metabolic activity of Saos-2 cultured on flat TCP versus electrospun scaffolds reveal distinct differences in cellular behavior. While OBs on TCP exhibit high rates of proliferation and metabolic activity, those on electrospun scaffolds display comparatively lower rates of these parameters. This phenomenon can be attributed to the inherent differences in the microenvironment provided by each culture system. For instance, a study demonstrated that human mesenchymal stem cells (MSCs) cultured on 3D-printed hydroxyapatite scaffolds exhibited enhanced proliferation and osteogenic differentiation compared to traditional culture methods, highlighting the importance of scaffold design in promoting favorable cellular responses [41].

As previously mentioned, ALP activity was significantly increased in Saos-2 cells cultured on TCP [42]. This increase in ALP activity corresponds with elevated gene expression levels of ALP, which were notably higher on days 7 and 14 in TCP cultures. These findings can be attributed to the role of ALP as an early marker of osteogenesis, as it is commonly used to assess the differentiation status of osteoblasts [43,44]. While cells on TCP proliferate rapidly, this fast growth may inhibit their ability to differentiate effectively compared to those seeded on 3D scaffolds. It was reported that rapid proliferation on flat surfaces can lead to reduced osteogenic potential, emphasizing the importance of scaffold design in promoting differentiation [45]. While our findings provide valuable insights, we recognize the limitation that ALP activity was not normalized to total protein content, a refinement that would enhance the quantitative interpretation of our results in future investigations. In contrast, cells cultured on fibrous scaffolds demonstrated an increase in mRNA expression of ALP by the final day of culture, indicating a greater capacity for osteogenic differentiation. This is consistent with studies that highlight the significance of ALP in monitoring OB function and mineralization processes during in vitro differentiation [46,47].

The expression of alkaline phosphatase (ALP) in osteoblasts is directly increased by osteoprotegerin (OPG) [48]. Our results align with this finding, as OPG expression peaked on day 21 in cells seeded on scaffolds, whereas it was not significantly elevated in TCP cultures. This suggests that the presence of scaffolds may enhance OPG expression, potentially due to the incorporation of HANPs in the nanofibers, which appears to support a dose-dependent increase in OPG levels. OPG plays a crucial role not only as a decoy receptor for RANKL but also as a marker for bone healing and turnover [49]. Studies have shown that OPG overexpression promotes OBs differentiation and enhances ALP activity, indicating its significant role in matrix maturation [50,51]. Furthermore, the regulation of key transcription factors such as Runx2 and Smad1 by OPG reinforces its importance in osteoblastogenesis [52]. These findings collectively support the hypothesis that OPG not only facilitates the differentiation of preosteoblasts into mature osteoblasts but also enhances ALP expression, thereby promoting effective bone formation.

OPG’s role is important in osteoclastogenesis as it prevents RANKL from binding to RANK receptors on monocytes and macrophages. In this manner, OPG naturally inhibits the expression of osteoclastic CTSK [53]. On the other hand, RANKL stimulates the gene and protein expression of CTSK in osteoclasts, which supports our results [54,55,56,57]. CTSK, a cysteine protease secreted by osteoclasts, is essential not only for collagen matrix degradation but also for the activation of TRAP [58,59]. Recent studies further elucidate the regulatory role of OPG in osteoclastogenesis. OPG acts as a decoy receptor for RANKL, effectively suppressing the maturation and activation of pre-osteoclasts by antagonizing RANK/RANKL signaling [60]. This regulation is crucial for maintaining bone homeostasis, as evidenced by findings that demonstrate enhanced osteoclast differentiation and function in the absence of OPG [61]. Moreover, OPG’s expression in osteoclasts themselves has been shown to play an autoregulatory role during late-stage osteoclastogenesis, where it influences both cell survival and resorptive activity [62]. These insights reinforce the importance of the OPG/RANKL/RANK axis in regulating bone resorption and highlight its potential as a therapeutic target for conditions characterized by excessive bone loss.

Herein, we demonstrated a connection between TRAP and CTSK mRNA, which were significantly more expressed in cells seeded on the scaffolds. CTSK and TRAP are also connected at the transcription factor level [63]. Conversely, the enzymatic activity of TRAP was relatively similar across all samples, regardless of whether they were cultured on scaffolds or TCP. These findings lead us to consider that TRAP activity may originate from undifferentiated monocytes/macrophages rather than solely from osteoclasts (OCs), due to deficiencies in gene expression of TRAP/CTSK. Notably, TRAP can be detected in cultures from undifferentiated cells right from the beginning. Interestingly, in cocultures grown on TCP, relative expression of RANKL was significantly lower than in those seeded on scaffolds by day 14, which may explain the reduced levels of expressed osteoclastic markers. RANKL acts as a regulator not only in osteoclastogenesis but also in osteoblast differentiation through RANK-RANKL reverse signaling. Previous findings suggest that RANKL present on osteoblasts exhibits reverse signal transduction ability [64]. Thus, the deficiency in mRNA levels of RANKL in cells seeded on TCP compared to their relative expression may clarify the excessive ALP expression observed in these cells. As RANKL is crucial for osteoblastic differentiation, its deficiency could impair maturation processes within these cells.

Moreover, the balance between RANKL and OPG is essential for maintaining bone homeostasis. Elevated levels of OPG can inhibit RANKL-mediated osteoclastogenesis, thereby reducing bone resorption [65]. This regulatory mechanism highlights the importance of scaffold design in modulating these signaling pathways. For instance, strontium-based bioactive scaffolds have been shown to enhance OPG expression while simultaneously reducing RANKL levels, promoting a favorable environment for OB differentiation [66]. Furthermore, studies indicate that mechanical stimuli can influence the expression of both RANKL and OPG, suggesting that optimizing scaffold properties could enhance bone regeneration by favoring OB activity over OC formation [67]. Understanding these interactions is critical for developing effective strategies aimed at treating bone-related disorders characterized by imbalances in bone remodeling.

Recent studies indicate that incorporating hydroxyapatite nanoparticles into PLCL scaffolds can modulate osteoblast/osteoclast (OB/OC) crosstalk by influencing the OPG/RANKL signaling axis. In OB/OC coculture systems, this composite scaffold environment affects the balance of signaling molecules, thereby regulating osteoclastogenesis and osteoblast differentiation. These findings support the idea that hydroxyapatite within PLCL matrices plays a crucial role in directing bone remodeling through biochemical cues in coculture models [68,69].

Additionally, collagen type I is produced exclusively by osteoblasts in the coculture. Procollagen is a form of collagen still present in the cell and later secreted to the extracellular space. Figure 7 shows collagen type I production by Saos-2 cells in coculture with THP-1-derived osteoclasts on PLCL scaffolds. Enhanced collagen type I production is observed on 7HA, followed by 3HA, with some protein detected on the pristine scaffold, but no signal on glass. Although signal intensity was not quantified, these images suggest that both the structure and amount of HA may stimulate collagen production. This aligns with previous research indicating that osteoblasts are the primary source of type I collagen in co-culture systems and that scaffold composition, including HA content, can influence osteoblast differentiation and matrix protein synthesis [70,71].

Building on these findings, our gene expression analysis further supports the immunostaining results, showing that COL1a1 expression was significantly higher on day 21 in cells cultured on 7HA scaffolds compared to 3HA and TCP. This elevated gene expression aligns with the observed increase in collagen type I protein production, suggesting that the higher HA content in the scaffold enhances osteoblast matrix synthesis. However, the presence of COL1a1 protein across all groups, despite lower gene expression in some, indicates that additional regulatory mechanisms or post-transcriptional processes may also contribute to protein synthesis and deposition. Notably, the expression patterns of RUNX2 and OCN—key markers of osteogenic differentiation—also increased on day 21 in all groups. RUNX2 is a master regulator of osteoblast differentiation, while OCN is associated with late-stage osteoblast maturation and mineralization [71,72]. The upregulation of these markers at the later time point may reflect a feedback loop driven by the dynamic interactions between osteoblasts and osteoclasts in the coculture system, as has been suggested in previous studies [73]. These findings highlight the importance of scaffold composition and cell–cell communication in regulating osteogenic gene expression and matrix protein synthesis, ultimately influencing bone tissue regeneration outcomes, and also emphasize the crucial role of cell–material interactions in directing osteoblast behavior and differentiation.

## 4. Methods

### 4.1. Scaffold Preparation

Scaffolds were prepared using a previously described method [27]. Briefly, poly(L-lactide-co-caprolactone) (PLCL, 70:30; PURASORB^®^ PLC 7015, Corbion, The Netherlands) was dissolved in chloroform to achieve a final solution concentration of 17% (*w*/*v*). Hydroxyapatite (HA) nanoparticles (Sigma-Aldrich, St. Louis, MO, USA; diameter less than 200 nm) were sonicated in acetone to form a nanoparticle suspension. These two solutions, along with varying concentrations of HA (0%, 3%, and 7% *w*/*v*), were mixed and stirred overnight to create the electrospinning solutions. The solutions were fed into a syringe and electrospun at a flow rate of 0.5 mL/h and a voltage of 15 kV. Fibers were collected on a rotating drum at a distance of 20 cm from the needle tip, with the temperature and humidity controlled at 16 °C and 80%, respectively.

### 4.2. Scanning Electron Microscopy (SEM)

The morphology of the electrospun fibers was analyzed using SEM (JEOL JSM-IT100, Tokyo, Japan/ Carl Zeiss Microscopy GmbH, Oberkochen, Germany). The samples were mounted on aluminum stubs with carbon tape and coated with a thin gold film for 60 s using a Cressington Sputter Coater 108 Auto (Cressington Scientific Instruments Ltd., Watford, UK). Fiber diameters were manually measured using Fiji/ImageJ v1.51 software (National Institutes of Health, Bethesda, MD, USA).

### 4.3. Cell Culture

- THP-1 Cells: Human THP-1 acute monocytic leukemia cells (ATCC, Manassas, VA USA) were grown in RPMI medium 1640 (Sigma Aldrich, St. Louis, MO, USA), supplemented with 10% heat-inactivated fetal bovine serum (FBS, Gibco, Grand Island, NY, USA; 10270-106) and 1% penicillin/streptomycin (P/S; Life Technologies, Carlsbad, CA, USA). The cells were cultured at 37 °C with 5% CO_2_.
- Saos-2 Cells: Saos-2 cells (ATCC, Manassas, VA, USA) were cultured in McCoy’s 5A medium (Merck, Darmstadt, Germany; M9309) supplemented with 15% fetal bovine serum (FBS, Gibco, Grand Island, NY, USA; 10270-106) and 1% P/S The cells were maintained in a humidified atmosphere with 5% CO_2_ at 37 °C.

### 4.4. Cell Seeding

Following established protocols for osteoclast differentiation, such as those described by Pasquier et al. (2017) and Li et al. (2017), we seeded THP-1 cells for macrophage differentiation [74,75]. THP-1 cells were seeded in 20 µL RPMI 1640 culture medium supplemented with 10% heat inactivated FBS and 1% P/S, and enriched with 100 ng/mL phorbol 12-myristate 13-acetate (PMA, Sigma-Aldrich, St. Louis, MO, USA, P8139-1MG), until Saos-2 addition, at a density of 1.5 × 105 cells per scaffold/glass or 5 × 104 per well in 96-well plates (TCP). After an initial 1 h adhesion, the medium was added to a final volume of 200 µL and left in an incubator with 5% CO_2_ at 37 °C for 3 days. After 72 h, Saos-2 cells were added to the culture at a density of 6 × 103 cells per scaffold/glass and 2 × 103 cells per well in 96-well plates in 40 µL for 1 h. The medium used was RPMI supplemented with growth factors—25 ng/mL macrophage colonies stimulating factor (MCSF, Peprotech, Rocky Hill, NJ, USA, 300-25) and 30 ng/mL ligand-receptor activator NF kappa B (RANKL, Peprotech; Rocky Hill, NJ, USA, 310-01). The medium was changed twice a week during the experiment.

### 4.5. Metabolic Activity

Metabolic activity was evaluated throughout the culture period using the MTS assay (CellTiter96^®^ AQueous One Solution Cell Proliferation Assay; Promega, Madison, WI, USA; G111A) on days 7, 14, and 21, (n = 6 samples per group). To perform the assay, the scaffolds were transferred into fresh 96-well plates, where the culture media was replaced with a mixture of 20 µL of MTS substrate and 100 µL of fresh media per well. Following a 2 h incubation at 37 °C and 5% CO_2_, the absorbance of the substrate was measured at 490 nm using a microplate reader (Infinite^®^ M200 PRO; Tecan Group Ltd., Männedorf, Switzerland).

### 4.6. Proliferation Analysis

Proliferation analysis was conducted on days 7, 14, and 21, (6 samples per group), using a fluorescence-based kit (Quant-iT™ PicoGreen^®^ dsDNA Assay Kit, Invitrogen Life Technologies, Carlsbad, CA, USA; Q33120). Following the MTS assay, cell lysis buffer (0.2% Triton X-100 Sigma-Aldrich, St. Louis, MO, USA; T8787; 10 mM Tris, Sigma-Aldrich, St. Louis, MO, USA; E1503; 1 mM EDTA; E6758 in distilled water) was added to the wells, followed by three freeze/thaw cycles with vortexing. Fluorescence intensity, measured using a microplate reader (λex = 485, λem = 535), allowed determination of DNA content from the kit’s calibration curve using 10 µL of cell lysate for the measurement.

### 4.7. ALP and TRAP Activity

The TRACP & ALP Assay Kit (Takara Bio Inc., Kusatsu, Japan; MK301) was utilized to simultaneously detect two key enzymes involved in bone metabolism: TRACP, an OC enzyme marker, and ALP, an OB enzyme marker on days 7, 14, and 21 (6 samples per group). This assay kit is designed for simple and rapid detection TRAP and ALP activities, employing pNPP (p-nitro-phenyl phosphate) substrate for enzymatic reaction. The scaffolds were transferred into new 96-well plates, where substrates were added and incubated according to the manufacturer’s instructions. Absorbance was measured at 405 nm using Tecan microplate reader. If the measured result exceeded the threshold, the sample was diluted with distilled water, and the final values were adjusted based on the dilution factor.

### 4.8. qPCR

RNA was extracted from samples (at least 3 samples per group) on days 7, 14, and 21 using the RNeasy Mini Kit (Qiagen, Hilden, Germany; 74104), following the manufacturer’s instructions. The RNA content and absorbance ratio at 260 nm to 280 nm were measured using the Tecan Infinite^®^ M200 Pro reader. Subsequently, cDNA synthesis was performed using the qScript cDNA Synthesis Kit (Quantabio, Beverly, MA, USA, 95047-500). Osteoblastic marker genes, including alkaline phosphatase (ALP), receptor activator of nuclear factor kappa B (RANKL), osteoprotegerin (OPG), Runt-related transcription factor 2 (RUNX2), osteocalcin (OCN) and collagen type I (COL1a1), along with the markers of osteoclasts, cathepsin K (CTSK) and tartrate-resistant acid phosphatase (TRAP), were analyzed. Fluorescence values for osteogenic genes were normalized by housekeeping gene GAPDH values. RT-PCR was carried out with the following parameters: activation at 95 °C for 10 min, amplification at 95 °C for 10 s and 60 °C for 10 s (for 50 cycles), and termination at 40 °C for 1 min. Applied Biosystems™ (TaqMan^®^, Thermofisher Scientific, Waltham, MA, USA; 4331182) probes were used for detection, and the Applied Biosystems™ TaqMan Gene Expression Master Mix (Thermofisher Scientific, Waltham, MA, USA; 4369510) was added to each sample. Graphs were generated from the calculated values using the formula 2−ΔCp. Samples were stored at −80 °C between RNA isolation, cDNA synthesis, and RT-PCR. Fluorescence intensity was measured using the Light Cycler 480 (Roche, Basel, Switzerland).

### 4.9. Dioc/Pi Staining

Cell distribution on the fibrous scaffolds was analyzed using confocal microscopy on days 7, 14, and 21 (triplicates per each group). Samples were fixed with frozen methanol at −20 °C for 10 min. After fixation, the fluorescent probe 3,3-diethyloxacarbocyanine iodide (DiOC6(3), 1 μg/mL in PBS, pH 7.4; Invitrogen, Waltham, MA, USA; D273) was added and incubated with the samples for 30 min at room temperature to visualize intracellular membranes. Next, cell nuclei were stained with propidium iodide (PI; 5 μg/mL in PBS, 5 min; Sigma Aldrich, Rocky Hill, NJ, USA; P4864), which intercalates into cell DNA. The scaffolds were then washed with PBS and imaged using a Zeiss LSM 880 Airyscan confocal microscope. The wavelengths used were λex = 488 nm and λem = 520 nm for DiOC6(3) and λex = 560 nm and λem = 580 nm for propidium iodide.

### 4.10. Collagen Type I Staining

For immunostaining, cells were fixed on day 21 with methanol (duplicates for each group), then permeabilized with 0.1% Triton™ X-100 (Sigma-Aldrich, St. Louis, MO, USA; T8787-100ML) containing 1% BSA for 30 min at room temperature. After PBS washes, samples were treated with 1% TWEEN^®^ 20 (Sigma-Aldrich, St. Louis, MO, USA; P9416-50ML) for 30 min, followed by additional PBS rinses. Pro-collagen type I primary antibody (1:20; DSHB, Iowa City, IA, USA; M-38-c) was applied in 1% BSA (Cytiva, Marlborough, MA, USA)/PBS and incubated overnight at 4 °C. After washing, Alexa Fluor 633-conjugated secondary antibody (2 μg/mL Invitrogen Life Technologies, Carlsbad, CA, USA; A21053) (1:2000) was added for 45 min at room temperature. F-actin was visualized with Phalloidin-iFluor-555 (1:400, Abcam, Cambridge, UK; ab176756), and nuclei were counterstained with Hoechst 33342 (1:500, 10 min; Invitrogen Life Technologies, Carlsbad, CA, USA; H21486). Samples were imaged using a Zeiss LSM880 Airyscan confocal microscope, and the wavelengths were as follows: for Phalloidin Ex/Em = 556/574 nm; AlexaFluor-633 Ex/Em = 631/650 nm; Hoechst 33342 Ex/Em = 361/497 nm.

### 4.11. Statistics

Statistical analyses were performed using GraphPad Prism 10.4.2 software (San Diego, CA, USA). The Shapiro–Wilk test assessed data normality. Normally distributed data were analyzed using ANOVA with Tukey’s post hoc test for multiple comparisons. Non-normal data were analyzed with the Kruskal–Wallis test (Dunn’s post hoc test where applicable). Columns represent group means ± standard deviation, with asterisks indicating statistical significance (*p* < 0.05).

## 5. Conclusions

Our study investigates biomaterials by comparing three types of PLCL scaffolds with varying amounts of HANPs and TCP. Specifically, these scaffolds were formulated with proportions of HANPs at concentrations of 7%, 3%, and 0%, respectively. Remarkably, our findings revealed substantial disparities between the structural attributes of the scaffolds and conventional tissue culture plastic substrates, prompting a deeper exploration into their implications for tissue engineering and regenerative medicine applications. Specifically, the scaffolds supported the interplay of the cells in a coculture of Saos-2 OBs and THP-1-derived OCs, mimicking bone physiology. Surprisingly, adding HANPs did not significantly enhance cellular responses, suggesting the need for further investigation. Our findings hold promise for advancing the field of biomaterials by contributing towards the development of a bone model suitable for applications such as drug testing and material evaluation. Continued research in this area has the potential to significantly enhance our understanding of bone biology and aid in the refinement of biomaterial-based approaches for various biomedical applications.

## Data Availability

The data presented in this study will be available at Zenodo repository.

## Abbreviations

The following abbreviations are used in this manuscript:

HANPs	Hydroxyapatite nanoparticles
ALP	Alkaline phosphatase
ACP5	Tartrate-resistant acid phosphatase type 5
P/S	Penicillin/Streptomycin
TCP	Tissue culture polystyrene
HA	Hydroxyapatite
OCN	Osteocalcin
TRAP	Tartrate-resistant acid phosphatase
RANKL	Receptor activator of nuclear factor κB ligand
ECM	Extracellular matrix
DNA	Deoxyribonucleic acid
qPCR	Quantitative polymerase chain reaction
PLA	Poly(lactic acid)
PCL	Poly(ε-caprolactone)
MTS	3-(4,5-dimethylthiazol-2-yl)-5-(3-carboxymethoxyphenyl)-2-(4-sulfophenyl)-2H-tetrazolium (cell viability assay)
RNA	Ribonucleic acid
PLGA	Poly(lactic-co-glycolic acid)
PLCL	poly(L-lactide-co-ε-caprolactone)
RPMI	Roswell Park Memorial Institute medium
